# Biochemical and Structural Characterization of a Novel Psychrophilic Laccase (Multicopper Oxidase) Discovered from *Oenococcus oeni* 229 (ENOLAB 4002)

**DOI:** 10.3390/ijms25158521

**Published:** 2024-08-05

**Authors:** Isidoro Olmeda, Francisco Paredes-Martínez, Ramón Sendra, Patricia Casino, Isabel Pardo, Sergi Ferrer

**Affiliations:** 1 Enolab, Departament de Microbiologia i Ecologia, Universitat de València, 46100 Burjassot, Valencia, Spain; isidoro.olmeda@uv.es (I.O.); sergi.ferrer@uv.es (S.F.); 2 Institut de Biotecnologia i Biomedicina (BIOTECMED), Universitat de València, 46100 Burjassot, Valencia, Spain; francisco.paredes@uv.es; 3 Departament de Bioquímica i Biologia Molecular, Universitat de València, 46100 Burjassot, Valencia, Spain; ramon.sendra@uv.es; 4 Group 739 of the Centro de Investigación Biomédica en Red sobre Enfermedades Raras (CIBERER) del Instituto de Salud Carlos III, 28029 Madrid, Spain

**Keywords:** psychrophilic laccase, biogenic amines, phenols, *Oenococcus oeni*, wine

## Abstract

Recently, prokaryotic laccases from lactic acid bacteria (LAB), which can degrade biogenic amines, were discovered. A laccase enzyme has been cloned from *Oenococcus oeni*, a very important LAB in winemaking, and it has been expressed in *Escherichia coli*. This enzyme has similar characteristics to those previously isolated from LAB as the ability to oxidize canonical substrates such as 2,2-azino-bis(3-ethylbenzothiazoline-6-sulfonic acid) (ABTS), 2,6-dimethoxyphenol (2,6-DMP), and potassium ferrocyanide K_4_[Fe(CN_6_)], and non-conventional substrates as biogenic amines. However, it presents some distinctiveness, the most characteristic being its psychrophilic behaviour, not seen before among these enzymes. Psychrophilic enzymes capable of efficient catalysis at low temperatures are of great interest due to their potential applications in various biotechnological processes. In this study, we report the discovery and characterization of a new psychrophilic laccase, a multicopper oxidase (MCO), from the bacterium *Oenococcus oeni*. The psychrophilic laccase gene, designated as LcOe 229, was identified through the genomic analysis of *O. oeni*, a Gram-positive bacterium commonly found in wine fermentation. The gene was successfully cloned and heterologously expressed in *Escherichia coli*, and the recombinant enzyme was purified to homogeneity. Biochemical characterization of the psychrophilic laccase revealed its optimal activity at low temperatures, with a peak at 10 °C. To our knowledge, this is the lowest optimum temperature described so far for laccases. Furthermore, the psychrophilic laccase demonstrated remarkable stability and activity at low pH (optimum pH 2.5 for ABTS), suggesting its potential for diverse biotechnological applications. The kinetic properties of LcOe 229 were determined, revealing a high catalytic efficiency (kcat/Km) for several substrates at low temperatures. This exceptional cold adaptation of LcOe 229 indicates its potential as a biocatalyst in cold environments or applications requiring low-temperature processes. The crystal structure of the psychrophilic laccase was determined using X-ray crystallography demonstrating structural features similar to other LAB laccases, such as an extended N-terminal and an extended C-terminal end, with the latter containing a disulphide bond. Also, the structure shows two Met residues at the entrance of the T1Cu site, common in LAB laccases, which we suggest could be involved in substrate binding, thus expanding the substrate-binding pocket for laccases. A structural comparison of LcOe 229 with Antarctic laccases has not revealed specific features assigned to cold-active laccases versus mesophilic. Thus, further investigation of this psychrophilic laccase and its engineering could lead to enhanced cold-active enzymes with improved properties for future biotechnological applications. Overall, the discovery of this novel psychrophilic laccase from *O. oeni* expands our understanding of cold-adapted enzymes and presents new opportunities for their industrial applications in cold environments.

## 1. Introduction

The two main groups of organisms involved in wine production are yeasts and lactic acid bacteria (LAB), which are responsible for alcoholic fermentation (AF) and malolactic fermentation (MLF), respectively. AF consists in transforming sugars into ethanol and carbon dioxide. During the MLF process, the L-malic acid present in wine is converted into L-lactic acid and carbon dioxide. MLF is a crucial step in winemaking as it enhances the organoleptic characteristics of wine and lowers the risk of microbial alteration. Among the LAB associated with the winemaking process, *Oenococcus oeni* is the species mainly responsible for MLF because it is the best-adapted bacterium to overcome wine stressing conditions, which is why it is often used as a malolactic starter culture [1]. Although this species is generally isolated from wine, it has occasionally been described in grape musts [2,3].

Enzymes play a fundamental role in numerous biological processes, and their unique properties have captivated researchers for decades. One intriguing aspect of enzyme functionality is their ability to operate efficiently under extreme conditions, including low temperatures. Enzymes that exhibit optimal activity at cold temperatures, known as psychrophilic enzymes, have garnered significant attention due to their potential applications in various biotechnological fields, ranging from industry to environmental bioremediation [4,5]. Among the psychrophilic enzymes, laccases, belonging to the multicopper oxidase family, have emerged as versatile catalysts with immense industrial potential. Laccases have been employed in the food industry to produce fruit juices and alcoholic beverages, including wine and beer. Laccases are frequently employed to prevent the oxidation of fruit juices. Fruit juices contain naturally occurring phenolics. The natural polymerisation and co-oxidation reactions of phenolics and polyphenols over time result in the development of undesirable changes in the colour and aroma of the juices. Some authors have employed laccase in conjunction with filtration to stabilise apple juice [6,7,8]. In the brewing industry, laccases have been employed to inhibit the formation of chill haze, which arises during the stabilisation stage and is caused by the interactions between haze active (HA) proteins and HA polyphenols. Additionally, the precipitation of proteins is triggered by proanthocyanidin polyphenols, which are present in small quantities. Laccase is capable of preventing haze formation by oxidising beer phenols [6,8,9]. Wine contains a high concentration of phenolics and polyphenols derived from the crushing, pressing, and maceration stages of production. These compounds contribute to the wine’s colour and astringency. The spontaneous oxidation of musts by oxygen and grape polyphenol oxidases results in alterations to the flavour profile and the development of browning. Laccases are employed to eliminate specific phenols and polyphenols that are responsible for the aforementioned issues [6,7]. In order to achieve the aforementioned objectives, it is essential that the laccases employed are capable of functioning at low temperatures, given that the stabilisation of juices, wines, and beers occurs at temperatures below 15 °C. Furthermore, laccases are also utilised in environmental bioremediation processes that take place in open air areas where temperature control is not feasible.

Laccases (benzenediol:oxygen oxidoreductase, p-diphenol oxidase EC 1.10.3.2) are blue multicopper oxidases (MCO), copper-containing enzymes that catalyse the oxidation of a substrate reducing molecular oxygen to water. Laccases are abundantly present in many plant, fungal, and bacterial species. These enzymes have found applications in diverse fields, such as pulp and paper production, textile dye degradation, wastewater treatment, and biosensor development. Laccases catalyse the monoelectronic oxidation of substrates at the expense of molecular oxygen and, generally, they have a broad range of substrates including phenols, such as methoxyphenols; polyphenols; and nonphenolic substrates, including aromatic amines, arylamines, anilines, thiols, and some cyanide complexes of metal [10]. Additionally, some laccases isolated from LAB are able to oxidize toxic substances as biogenic amines (BA) [11]. Laccases can expand their substrate range through the use of redox mediators, which are low molecular weight molecules that are able to shuttle electrons between laccases and target molecules that otherwise could not be oxidized [10].

Our group have performed studies on the structures of some of LAB laccases in *Pediococcus pentosaceus* (Pp4816) and *P. acidilactici* (Pa5930) from fermented products, which exhibited an extended N-terminal and an extended C-terminal rich in Met end as well as two conserved Met residues at the entrance of the T1Cu site [11].

Traditional laccases derived from mesophilic sources typically operate optimally at moderate to high temperatures, which limits their applicability in cold environments or processes that require low-temperature conditions. The discovery and characterization of psychrophilic enzymes have opened up new possibilities for enzymatic transformations under cold conditions. Psychrophilic enzymes have evolved unique structural and functional adaptations to maintain their activity and stability at low temperatures, and understanding these adaptations can provide valuable insights for industrial biocatalysis [12]. In particular, psychrophilic laccases offer distinct advantages over their mesophilic counterparts, including enhanced activity at low temperatures, increased catalytic efficiency, and improved stability in cold environments [13].

Previously, other alleged ‘psychrophilic’ laccases have been described but none with real activity at such low temperatures: The laccase from *Cryptococcus albidus* (syn. *Naganishia albida*) showed maximum activity at 20–30 °C [14]. Later, Rovati*,* et al. [15] noted the laccase activity at 15 °C from 25 yeast strains isolated from soil samples from de Mayo/King George Island (Antarctica). Roulling, et al. [16] described an optimal temperature around 35 °C for the laccase of the Antarctic bacterium *Pseudoalteromonas haloplanktis* (PhaMOx). The laccase from the fungus *Kabatiella bupleuri* G3 from the Italian collection, Industrial Yeasts Collection DBVPG (Perugia, Italy), showed an optimum temperature for enzyme operation in the range of 30–40 °C [17], with an optimal temperature of 30 °C for its purified recombinant laccase [18]. The heterologously expressed MCO from *Lactilactobacillus sakei*, which has not been described as psychrophilic, showed an optimal temperature of 25 °C [19]. Recently, a novel laccase from the psychrotolerant Antarctic marine *Halomonas* sp. strain M68 was described [20]; however, its optimal temperature was in the 40–50 °C range.

The primary objective of this study was to identify, isolate, and characterize a novel psychrophilic laccase from *Oenococcus oeni*. Through genomic analysis, recombinant expression, biochemical characterization, and structural analysis, we aim to unravel the molecular mechanisms underlying the psychrophilic adaptation of this laccase. Furthermore, we have investigated its substrate specificity, catalytic efficiency, and stability profile to evaluate its potential for industrial applications in cold environments.

By gaining a deeper understanding of the unique properties and functional characteristics of psychrophilic laccases, we can expand the biocatalytic toolbox and pave the way for the development of novel enzyme-based processes that can operate efficiently at low temperatures. This research not only contributes to our knowledge of cold-adapted enzymes but also opens avenues for the exploitation of psychrophilic enzymes in biotechnology and other fields requiring low-temperature enzymatic transformations.

## 2. Results

### 2.1. Isolation and Sequence Analysis of the Laccase Gene of the O. oeni 229

The amplification with the primer forward LcOe1 and the reverse primer LcOe2 of the total *O. oeni* 229 DNA rendered a fragment of 1641 bp that codified for a putative multicopper oxidase (GenBank code PQ112773), homologous to the corresponding gene in the *O. oeni* PSU-1 genome (CP000411.1).

**Figure 1:**
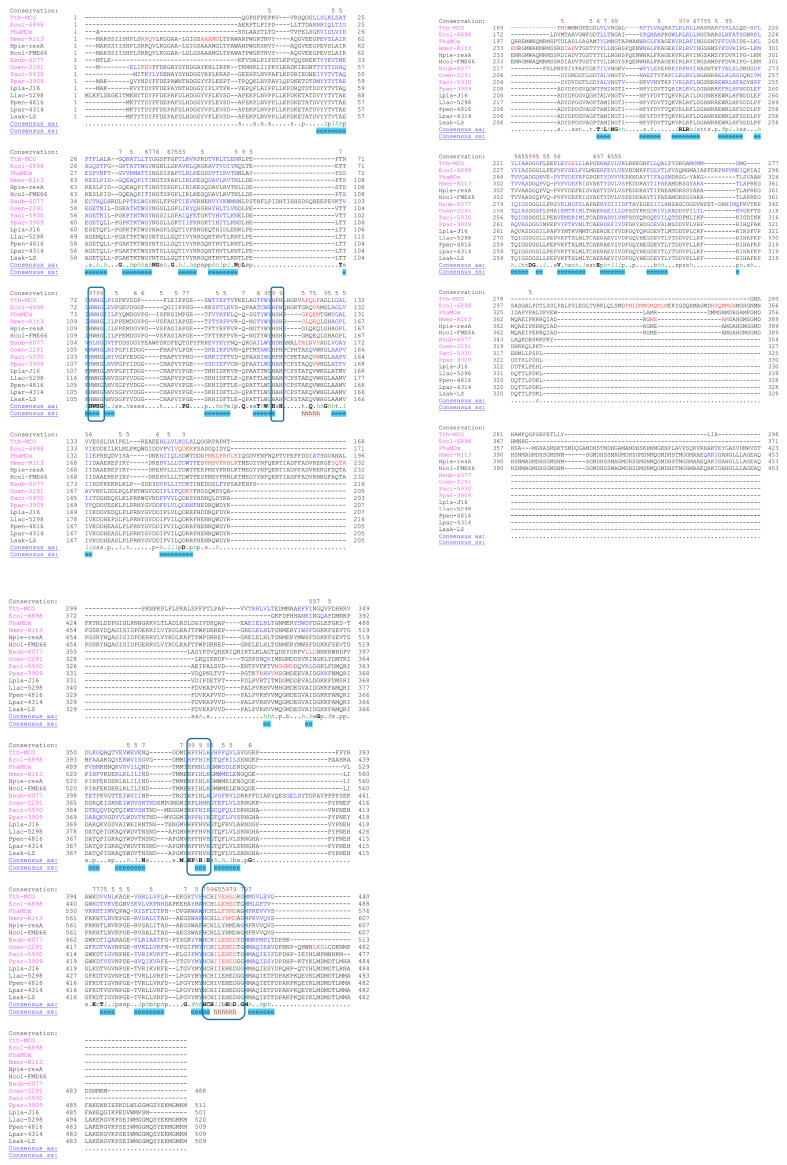
Multiple sequence alignments and conservation of laccases from psychrophilic, mesophilic, and thermophilic organisms is shown. Scoring of the Hmer-R1t3 (*Halomonas meridiana* CopA), Hpie-resA (*Halomonas piezotolerans*), Hcol-FME66 (*Halomonas colorata*), PhaMOx (*Pseudoalteromonas haloplanktis*), Ecol-6898 (*Escherichia coli* CueO), Bsub-6077 (*Bacillus subtilis* CotA), Ppar-3909 (*Pediococcus parvulus*), Llac-5298 (*Lactococcus lactis*), Ppen-4816 (*Pediococcus pentosaceus*), Lpar-4314 (*Lacticaseibacillus paracasei*), Lpla-J16 (*Lactiplantibacillus plantarum*), Paci-5930 (*Pediococcus acidilactici*), Lsak-LS (*Latilactobacillus sakei*), Ooen-2291 (*Oenococcus oeni* LcOe 229), and Tth-MCO (*Thermus thermophilus*) laccases was performed using Promals3D software (http://prodata.swmed.edu/promals3d/promals3d.php, accessed on 18 May 2024) [23,24]. The sequence of Paci-5930 is representative of the shortest C-terminus in LAB. The first row in each block shows conservation indices for positions with a conservation index greater than 4. The last two rows show the consensus amino acid sequence (Consensus_aa) and consensus-predicted secondary structures (Consensus_ss). Representative sequences have magenta names and they are coloured according to predicted secondary structures (red: alpha-helix, blue: beta-strand). The sequences with black names directly under a representative sequence are in the same pre-aligned group and are aligned in a fast way. Consensus predicted secondary structure symbols: alpha-helix: h; beta-strand: e. Consensus amino acid symbols are: conserved amino acids are in bold and uppercase letters; aliphatic (I, V, L): *l*; aromatic (Y, H, W, F): *@*; hydrophobic (W, F, Y, M, L, I, V, A, C, T, H): *h*; alcohol (S, T): o; polar residues (D, E, H, K, N, Q, R, S, T): p; tiny (A, G, C, S): t; small (A, G, C, S, V, N, D, T, P): s; bulky residues (E, F, I, K, L, M, Q, R, W, Y): b; positively charged (K, R, H): **+**; negatively charged (D, E): **-**; charged (D, E, K, R, H): c. Dashes indicate gaps to maximise alignment. The motifs forming the four copper ligands, which are highly conserved in laccases (conserved sequences of these motifs are HXHG, HXH, HXXHXH and HCHXXXHXXXXM/L/F), are enclosed in boxes [25].

Protein sequence analysis revealed the four conserved copper ligand motifs characteristic of the MCO family [21] (Figure 1). Alignments with other laccases from psychrophilic, mesophilic, and thermophilic organisms showed a high conservation of the four typical motifs of these MCOs (Figure 1) but also large differences in some regions representing typical external loops found in some of these enzymes.

### 2.2. Cloning, Expression, and Purification and Characterization of the Laccase LcOe 229

LcOe 229 yields from *E. coli* BL21(DE3), grown in 2 L LB + kanamycin + chloramphenicol + CuCl_2_, were 297.69 mg of crude total protein from which 3.71 mg corresponded to the purified laccase (3% yield from total protein).

**Figure 2:**
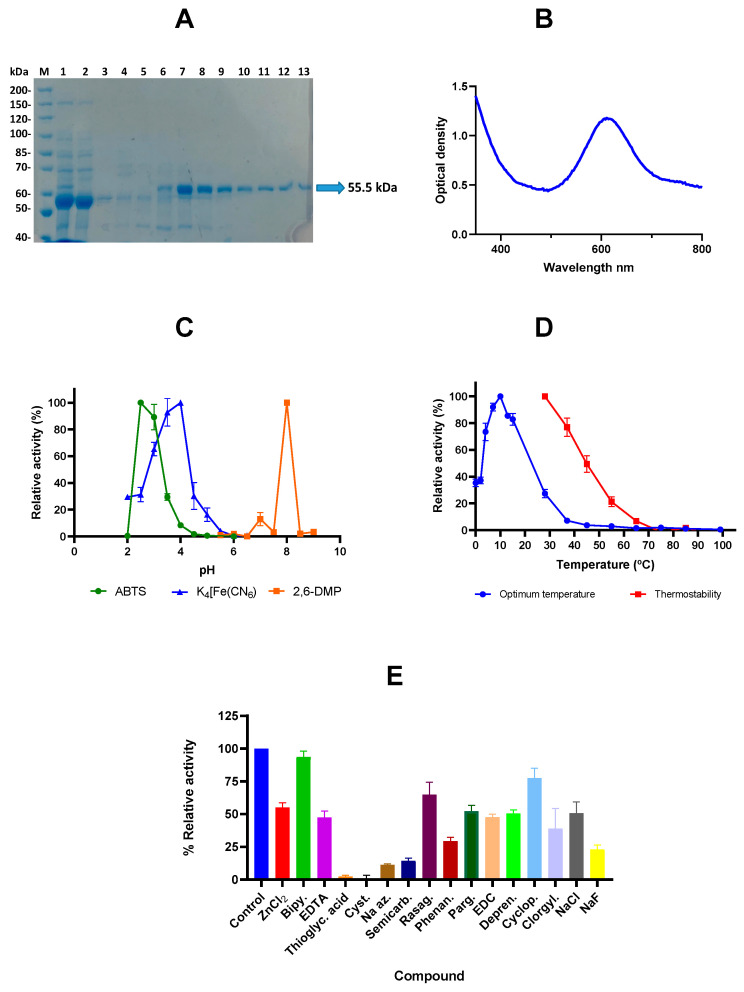
Purification and characterization of the recombinant protein LcOe 229. (**A**) PageBlue-stained 7.5% SDS-polyacrylamide gel electrophoresis of the different steps of the purification process. Lane M, page ruler unstained marker (Thermo Scientific). Lane 1: whole-cell extract from post-induction cells; lane 2: unbound proteins collected in the flowthrough; lane 3: proteins collected after washing with 5 mM imidazole buffer; lane 4: proteins collected after washing with 20 mM imidazole buffer; lanes 5–13: successive fractions of the elution from the metal-chelating chromatography on Ni^2+^-NTA-agarose; arrow marks recombinant protein. (**B**) Absorption spectrum of the purified protein. (**C**) Optimum pH for the oxidation of the substrates 2,2-azino-bis(3-ethylbenzothiazoline-6-sulfonic acid) (ABTS), 2,6-dimethoxyphenol (2,6-DMP), and potassium ferrocyanide K_4_[Fe(CN_6_)], of the LcOe 229 laccase. (**D**) Optimum temperature and thermostability of the LcOe 229 measured using ABTS as substrate. (**E**) Effect of different putative inhibitors on the laccase LcOe 229. ZnCl_2_: zinc chloride; Bipy.: 2,20-bipyridyl; EDTA: ethylenediaminetetraacetic acid; *Thioglyc.* acid: thioglycolic acid; *Cyst.*: cysteine HCl monohydrate; Na Az.: sodium azide; Semicarb.: semicarbazide; Rasag.: rasagiline; Phenan.: 1,10-phenanthroline; Parg.: pargyline; EDC: N-(3-dimethyl aminopropyl)-N′-ethyl carbodiimide; Depren.: deprenyl; Cyclop.: cyclopropenyl; Clorgyl.: clorgyline; NaCl: sodium chloride; NaF: sodium fluoride; control means an enzyme reaction in absence of inhibitors. The remaining activity with the different potential inhibitors is graphed as percentage respect to the control (assumed as 100% of enzyme activity). Values are means ± standard deviations of triplicate assays.

The purification process rendered an excellent good quality protein, as can be seen in Figure 2A. The purified protein had a molecular mass of 55.5 kD (Figure 2A), with an isoelectric point of 5.70 and a 4.1% methionine in the amino acid composition. It showed the two typical absorption peaks at 330 and 600-610 nm of the blue laccases [22] (Figure 2B).

The determination of the kinetic parameters of recombinant LcOe 229 for the 2,2-azino-bis(3-ethylbenzothiazoline-6-sulfonic acid) (ABTS), 2,6-dimethoxyphenol (2,6-DMP), and potassium ferrocyanide K_4_[Fe(CN_6_)] was achieved by monitoring the increase in OD_420_ over time at different substrate concentrations. The experimental curve of initial rates against ABTS concentration was slightly sigmoidal, but the sigmoidal kinetic profile was only observed in this substrate and not with 2,6-DMP and ferrocyanide (Supplementary Figure S1), which fitted to hyperbolic curves. The kinetic parameters for these three substrates are shown in Table 1. The Km (K_0.5_) for ABTS was ten and seventeen times lower than that of 2,6-DMP and ferrocyanide, whereas the K_cat_ were very similar for ABTS and ferrocyanide, but one hundred times lower for 2,6-DMP, which indicates that is it not a good substrate for the enzyme at the conditions used.

The optimum pH of this laccase depended on the substrate to be oxidised, so for ABTS and potassium ferrocyanide, the optimum pH was acidic, 2.5 and 4, respectively (Figure 2C). The ABTS-oxidising activity decreased to 0% below 2.5 and to 30% above pH 3, whereas for potassium ferrocyanide, the activity decreased to 65% at pH 3.5 and to 30% at pH 4.5. The optimum pH for 2,6-DMP was 8, and the activity of the enzyme decreased to 0% below or above this pH.

The optimum temperature on ABTS was as low as 10 °C, although percentages of activity above 70% were maintained between 4 and 15 °C, but at temperatures of 28 °C or higher the activity dropped below 30% (Figure 2D). This enzyme showed poor thermal stability, as a 50% drop of relative activity occurred when the enzyme was incubated at 45 °C for 5 min (Figure 2D).

Significant differences (*p* ± 0.0001) were found in the effect of the putative inhibitory compounds (Figure 2E). Cysteine and thioglycolic acid completely inhibited the ABTS-oxidizing activity of LcOe 229. Semicarbazide and sodium azide decreased the enzyme activity to less than 15%. Phenanthroline and sodium fluoride lowered the activity to 30% or less.

A decrease from 39 to 55% was observed with ZnCl_2_, EDTA, pargyline, EDC, deprenyl, clorgyline, and NaCl. Rasagiline and cyclopropylamine decreased the activity to 65 and 77%, respectively. The less inhibitory compound was 2,2′-bipyridyl, which maintained a 94% residual activity.

### 2.3. Structure of LcOe 229

**Figure 3:**
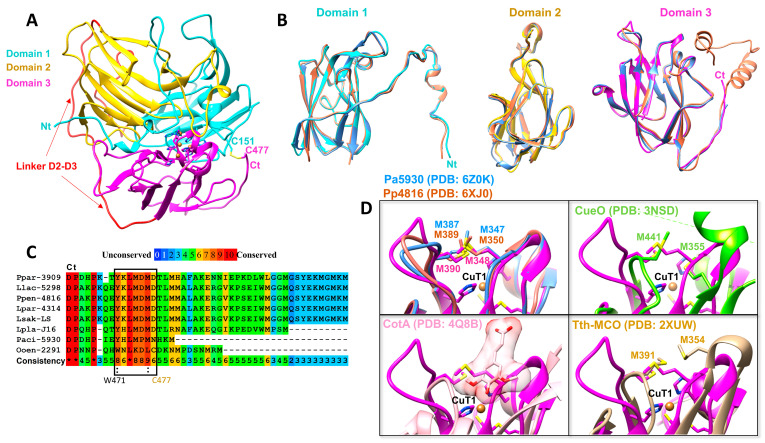
Structure of LcOe 229 compared with other laccases. (**A**) Cartoon representation of LcOe 229 highlighting each domain by colour-coding (Domain 1 in cyan, Domain 2 in yellow, and Domain 3 in magenta) and the linker D2–D3 in red. (**B**) Superposition of each domain colour-coded as in (**A**) with the structure of LAB laccases Pa5930 (in blue) and Pp4816 (in orange). (**C**) Sequence and structural alignment of the C-terminal end for LcOe 229 with several LAB laccases Ppar-3909 (*P. parvulus*), Llac-5298 (*L. lactis*), Ppen-4816 (*P. pentosaceus*), Lpar-4314 (*L. paracasei*), Lpla-J16 (*L. plantarum*), Paci-5930 (*P. acidilactici*), Lsak-LS (*L. sakei*). Framed is a rather conserved region in LAB which presents more differences in LcOe. The scoring scheme works from 0 for the least conserved alignment position, up to 10 for the most conserved alignment position, and the identical conserved residues are indicated by asterisks (colour scale indicated on top). (**D**) Superposition of Domain 3 (in magenta) from LcOe 229 with different structures of laccases Pa5930 (in blue; PDB: 6Z0K), Pp4816 (in orange; PDB: 6XJ0), CueO (in green; PDB: 3NSD), CotA (in pink; PDB: 4Q8B), and Tth-MCO (in light brown; PDB: 2XUW). Side chains for the two Met (M348 and M390) at the entrance of T1Cu site of LcOe 229 are shown as well as the side chains of two Met at a similar site found in other laccases. For CotA, it is shown the substrate sinapic acid which lies on the Met.

To study the structural features of LcOe 229, we determined its crystal structure (Figure 3). Data collection and refinement statistics are included in Table 2. We obtained two types of crystals for LcOe 229, one for the enzyme without Histag and the other for the enzyme with Histag, that were grown under two different conditions and which diffracted X-rays to 3 and 3.5 Å, respectively, each one having a different monoclinic space group (Table 2). The asymmetric unit of each crystal contained two molecules, which exploited two packing interfaces; however, according to the PISA server, both interfaces were the result of crystal packing (Supplementary Figure S2A). To ensure the biological oligomeric state of LcOe 229, we performed size exclusion chromatography (SEC) analysis which indicated that it was monomeric (Supplementary Figure S2B). A comparison of the two molecules in the asymmetric unit of each crystal indicated that they were identical, even between both crystal types (RMSD between molecules was 0.2 Å for 476 residues), demonstrating absence of conformational changes between them.

The sequence of LcOe 229 shares a relatively high sequence identity with the laccases of Pp4816 from *Pediococcus pentosaceus* and Pa5930 from *P. acidilactici* (53.5% and 56.4%, respectively), the structures of which have been determined previously by our group [11]. The structure of LcOe 229 shows a similar fold to Pp4816 (PDB: 6XJ0; RMSD of 1.1 Å for 459 residues) and Pa5930 (PDB: 6Z0K of 0.9 Å for 457 residues), formed by three cupredoxin-like domains (D1, D2, and D3; Pfam CL0026) (Figure 3A) with high structural similarity to Pp4816 and Pa5930, only observing differences in the linker regions that connect the three domains (linker D1-D2 and linker D2-D3) (Figure 3B and Supplementary Figure S3). The structures of Pp4816 and Pa5930 revealed three features that were rather specific to LAB laccases. They involved an extended N-terminal, an extended C-terminal end with respect to CotA from *B. subtilis* and CueO from *E. coli* rich in His and Met residues, and two Met residues at the substrate binding site observed for CotA close to the T1Cu site [11].

The structure of LcOe 229 showed the three specific structural features observed in the LAB laccases. Domain 1 had the extended N-terminal (residues 1 to 170) and Domain 3 had the extended C-terminal end (residues 466 to 488) which was eight residues longer than Pa5930 and contained one His (H470) and three Met (M481, M486, and M488) but was shorter than other LAB laccases (Figure 3B,C). Interestingly, in both crystals of LcOe 229, we just could trace the C-terminal end until residue C477, which was forming a disulphide bond with residue C151 of Domain 1 (Figure 3A). There was no electron density for the last eleven residues, an indication of disorder and flexibility. In comparison with other LAB laccases, the C477 located at the C-terminal is just present in *O. oeni* (Figure 3C); however, the position of C151 is conserved. Moreover, in Pp4816, C151 is forming a disulphide bond with C121 at Domain 1, a Cys that is conserved in other LAB laccases except in Pa5930 and LcOe 220 which is a Pro (Figure 1). Thus, we could envision the formation of a disulphide bond in Domain 1 between C121 and C151 for other LAB laccases as well.

The C-terminal of LcOe showed the lowest sequence identity (~29%) compared with other LAB laccases. Indeed, we found residues conserved in LAB laccases which differed in LcOe 229, such as K474 instead of Met, W471 instead of Tyr, and L476 instead of Met, albeit the nature of the last two residues was conserved (Figure 3C). In relation to the conserved Met residues at the T1Cu site, LcOe 229 showed M348 and M390 in Domain 3 at the same position as in Pp4816 and Pa5930, thus blocking the binding site of substrates bound to CotA exemplified by sinapic acid (PDB: 4Q8B) [26] (Figure 3D), or other fungal laccases such as MaL from *Melanocarpus albomyces* [27]. Interestingly, these two Met residues lie almost as two other Met residues that belong to the Met-rich insert of CueO (M355 and M441; PDB: 3NSD). Also, the laccase Tth-MCO from *T. thermophilus* contains two Met residues (M354 and M391; PDB: 2XUW) which are close to its Met-rich β-hairpin. Moreover, the two Met from Tth-MCO were involved in binding the metal ion Ag^+^ [28], and the two Met in CueO have been found to bind Ag^+^ and Cu^+^, revealing a new substrate binding site [29]. In this way, we propose that the two Met of LcOe 229 could have a role in metal ion binding as a substrate binding site (Figure 3D).

### 2.4. Organization around the Copper Binding Sites of LcOe

**Figure 4:**
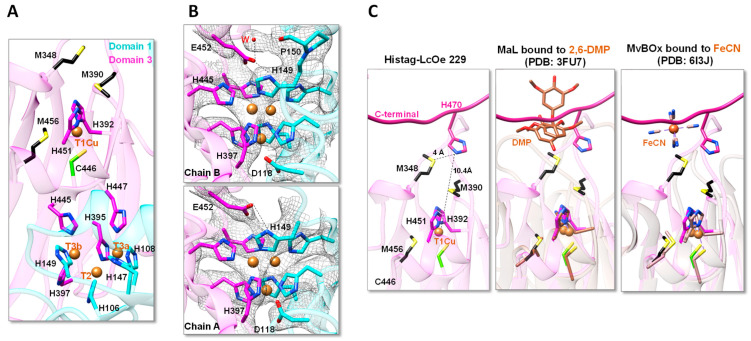
Organization of the copper binding sites in LcOe 229. (**A**) Representation of the configuration observed for T1Cu and TNC showing the side chain of the residues coordinating the copper ions (in orange). The His residues are color-coded as Domain 1 (in cyan) and Domain 3 (in magenta) while Cys are colored in green and Met are colored in black (sulfurs are in yellow). The two Met at the entrance of T1Cu are also shown. (**B**) Zoom of the TNC site in chain B and in chain A of LcOe 229 showing the electron density of 2fo-fc map at 1σ. The side chains of the His coordinating the coppers are color-coded as the domains. The E452 and D118 involved in proton coordination are also shown. (**C**) Structure of Histag-LcOe 229 showing how the C-terminal of one molecule (Domain 3 in magenta) is in close distance to the Met residues (M348 and M390) at the entrance of T1Cu from the other molecule (Domain 3 in transparency magenta). The central and right panel show a superposition of Histag-LcOe 220 with *M. albomyces* laccase (MaL) in the presence of 2,6-DMP (PDB: 3FU7) and bilirubin oxidase from *M. verrucaria* in the presence of ferricyanide (PDB: 6I3J). Dotted lines are an indication of distance between atoms and distance values are also shown.

The structure of LcOe 229 showed the T1Cu site with a Cu^2+^ ion coordinated by two His (H451 and H392), one Cys (C446), and one Met (M456). Meanwhile, the TNC contained three copper ions (T3a, T3b, and T2) coordinated by eight His residues. Specifically, T2 is coordinated by H106, H395, and H397; T3a is coordinated by H108, H147, and H447; and T3b is coordinated by H149, H397, and H445 (Figure 4A). The electron density around the His residues of the TNC in a 2F_0_-F_c_ map (at 1 σ) does not show a positive peak for O_2_ or H_2_O/OH^−^ molecules at the entry or exit channel, although positive electron density can be observed in a difference map. Coordinated to the His residues at the TNC, LcOe 229 showed the two conserved acidic residues, D118 and E452, which have been involved in proton-relay for the reduction of O_2_ to H_2_O molecules [30]. Residue D118 interacts with H397 close to the exit channel; however, its side chain is not binding any water molecule that should stabilize the H_2_O/OH^−^ molecules produced by O_2_ reduction and which should be coordinated with T2 (Figure 4B). Also, in one of the molecules (chain B), there is no electron density for the carboxylate group of E452, but the most plausible rotamer interacts with H149 and a water molecule whose electron density is connected to the main chain oxygen of P150 (Figure 4B). Meanwhile, in the other molecule (chain A), there is electron density for the carboxylate group of E452, which is connected to a grown electron density that would accommodate a water molecule, which interacts with H149. In this way, E452 shows flexibility between both molecules to carry out its function, as has been proposed in Tth-MCO [30]. Overall, the absence of O_2_ or H_2_O molecules at the entry or exit of the TNC could be the result of low structural resolution and radiation damage.

As we mentioned previously, the two conserved Met at the entrance of the T1Cu site (M348 and M390) found in LAB laccases may have a role in substrate binding (Figure 4C). Surprisingly, in the Histag-LcOe 229 structure determined at 3.5 Å, the residue H470 at the C-terminal end of one molecule is in close distance (3.9 Å) of M348 from a symmetric molecule. Thus, we propose that the His side chain could emulate the position of a plausible substrate, such as 2,6-DMP or ferricyanide ion (FeCN) (Figure 4C). Indeed, we have found that in the structure of the recombinant laccase MaL from *M. albomyces* bound to 2,6-DMP and its dimeric product (PDB: 3FU7), the substrate and product locates in a binding site close to H470 [27]. Moreover, the structure of the bilirubin oxidase MvBOx from *Myrothecium verrucaria* shows a binding site for FeCN close to H470 (PDB: 6I3J) [31]. The distance between these substrates and T1Cu is rather large (Nε2 of H470 is at 10.4 Å of T1Cu); however, in the catalysis carried out by laccases, the electronic transfer is performed at very large distances [28]. This is also the case for MvBOx, in which the shortest distance between FeCN and T1Cu is 11.4 Å, which is within the natural 4 to 14 Å range used for electron tunneling [32].

### 2.5. Comparison among LcOe 229 and Other Laccases

Laccases from Antarctic psychrophilic bacteria have been identified, such as PhaMOx (from *P. haloplanktis*) and Ant from *Halomonas* sp. strain M68, which shares sequence identity with CopA from *H. meridiana* and multicopper oxidases from *H. piezotolerans* and *H. colorata* FME66 [20,33]. A primary structure analysis of Antarctic laccases PhaMOx and *Halomonas* sp. indicates a decrease in their aliphatic index (~74%) compared with CueO (83%) and Tth-MCO (97%) correlating with an increase in adaptation temperature with increased stability through the hydrophobic effect [16]. Now, we have calculated the aliphatic index for the psychrophilic LcOe 229, which was 81%, a higher value than the Antarctic laccases and other LAB laccases (~73%) but closer to CueO (Table 3). The fact that the mesophilic and LAB laccases show an aliphatic index similar to Antarctic laccases indicate that the adaptation to temperature does not seem to be directly correlated with the aliphatic index.

Also, it has been proposed that psychrophilic proteins cluster Gly residues and reduce Pro residues in loops to increase flexibility, as well as reduce Arg residues to decrease the number of salt bridges. Moreover, they reduce the number of ion pairs and hydrogen bonds compared with mesophilic enzymes and their pI is frequently more acidic than in mesophilic [34]. We have analysed the primary structure content of laccases from the Antarctic bacteria previously mentioned compared with LcOe 229, mesophilic (CueO and CotA), thermophilic (Tth-MCO), and several LAB laccases to find correlations between sequence and activity (Table 3). The pI for LcOe 229 was 5.7, a similar value in LAB laccases (~5.3), Antarctic laccases (~5.6), and mesophilic (~6), except for Tth-MCO, which was increased to 7.1, thus no correlation was observed between the enzymatic optimal temperature and pI. The relative content of Gly residues was 10% in *Halomonas* sp. laccases and CueO, but it was around ~8% for the rest of the laccases except for CotA, which was even lower (6.6%). Meanwhile, the Pro content was ~5.5% in Antarctic laccases, which increased to ~7.4% for LAB laccases, CueO, and PhaMOx and up to 10% for CotA and Tth-MCO. Interestingly, the Arg content was ~7.5% in *Halomonas* sp. laccases, CueO, and Tth-MCO but dropped to ~5.5% in PhaMOx and CotA and to ~3.8% in LAB laccases including LcOe 229. In line with the increase in Arg content, there was an increase in Glu at the *Halomonas* sp. laccases and Tth-MCO (~7.4%) versus LAB laccases (5.6%) and PhaMOx (4.5%). Contrary to Glu, the additional acidic residue Asp was increased in LAB laccases and PhaMOx (~8.5%) versus *Halomonas* sp. laccases and mesophilic (~5.9%). In relation to the polar residues Asn and Gln, their content was similar in Antarctic and LAB laccases (~3.2 and ~3.5%, respectively) and a reduced content in Asn and Gln in Tth-MCO was observed (Table 3).

In this way, as predicted for psychrophilic proteins that increase flexibility, the Antarctic laccases from *Halomonas* sp. had a higher content of Gly and a reduced content of Pro, but their Arg and Glu content was higher than expected. Meanwhile, the Antarctic PhaMOx showed a residue content similar to LcOe 229 and other LAB laccases in terms of Gly, Arg, Glu, and Asn, an indication that the adaptation to temperature did not correlate with the enzymatic optimal temperature. Indeed, even between different LAB species, some laccases showed enzymatic optimal temperatures at ~27 °C while others showed this at ~58 °C.

Interestingly, the Antarctic laccases showed a ~6% content of Met similar to CueO, a slightly higher value than LcOe 229 and LAB laccases (~4%) compared with Tth-MCO and CotA (Table 3). In contrast, the Cys content was low in Antarctic laccases (0.2%) compared with LAB laccases (Table 3). For the Antarctic laccases, the highest Met content was found at a very long linker D2-D3 in Domain 2, which has been compared with the Met-rich insertion of CueO, and which has been demonstrated to be important for phenol-oxidase activity and additional copper binding in PhaMOx [35]. We have modelled the structures of the Antarctic laccases with AlphaFold and these insertions were not present in LcOe 229 or in an AlphaFold model of *L. sakei* (Supplementary Figure S4).

Finally, the difference in amino acid content between the laccases under study together with differences in the alignments (Figure 1) were consistent with the phylogenetic distribution of these enzymes where the Antarctic laccases diverged from LcOe 229 and the mesophilic laccases (Supplementary Figure S5). Moreover, LcOe 229 was more similar to Pa5930 than to other LAB laccases. Overall, the psychrophilic activity of LcOe 229 cannot be explained by any similarities with the Antarctic laccases either at primary or tertiary structural levels.

## 3. Discussion

LcOe 229 laccase has shown a sigmoidal kinetic behavior on ABTS and Michaelis–Menten kinetics on 2,6-DMP and ferrocyanide, in a similar way as other LAB laccases previously described [11,36]. The K_0.5_ of LcOe for ABTS was lower than the value of Pp4816, Pa5930, and *L. paracasei* ENOLAB 4314, but was higher than the value of *P. parvulus* ENOLAB 3909 and *Lactococcus lactis* 5298 [36]. The Km for 2,6-DMP was lower than the observed value in Pp4816 and Pa5930, but for ferrocyanide, it was a similar value in Pa5930, although higher than in Pp4816. The Kcat on ABTS was similar to that of Pp4816, although higher than that of Pa5930 and of the three laccases described by Monroy, Olmeda, Ferrer, and Pardo [36]; however, the Kcat value for 2,6-DMP was similar to those described by Olmeda, Casino, Collins, Sendra, Callejón, Huesa, Soares, Ferrer, and Pardo [11] but lower for ferrocyanide. The optimum pH for ABTS oxidation was similar to that of *P. parvulus* ENOLAB 3909 and lower than those of Pp4816 and Pa5930 laccases [10,11,36,37]. The most singular feature of LcOe 229 is its optimal temperature, which is fairly low, compared not only with other LAB laccases but with that of the laccase PhaMOx isolated from the Antarctic bacterium *P. haloplanktis* [10,22,36,37].

The amino acid sequence analysis performed on some LAB laccases (Table 3 and Supplementary Figure S5) has revealed a high similarity between LcOe 229 and LAB laccases Pa5930, Pp4816, and those of the *Lactobacillus plantarum* J16, *Pediococcus parvulus* ENOLAB 3909, *Lacticaseibacillus paracasei* ENOLAB 4314, and *Lactococcus lactis* ENOLAB 5298, previously characterized by our group, and with other LAB. In the phylogenetic study performed by Olmeda, Casino, Collins, Sendra, Callejón, Huesa, Soares, Ferrer, and Pardo [11], the laccase of *O. oeni* 8404 was placed in the same group as *Pediococcus acidilactici* 7910, *O. kitaharae* 7974, *L. argentinicus* 50539, etc.; this group exhibited a shorter C-terminus than laccases of other LAB species as *P. pentosaceus* 4816. Now, in the phylogenetic tree that we have generated based on laccases from psychrophilic, mesophilic, and thermophilic bacteria (Supplementary Figure S5 [38,39]), it can be shown that the most proper neighbor of LcOe 229 is that of *Pediococcus acidilactici* (Paci-5930); it exhibited an optimal temperature of 28 °C and retained more than 95% activity at 20 °C. At a greater distance from LcOe 229 are the laccases CueO from *E. coli* and CotA from *B. subtilis*, bacteria recognised as mesophilic, although the optimum temperature of their laccases is high enough to be considered thermophilic. However, LcOe 229 is far away not only from the thermophilic Tth-MCO from *T. thermophilus*, as would be expected, but from PhaMOx from the psychrophilic Antarctic bacterium *P. haloplanktis*.

When we compared the aminoacid content and chemical parameters of some psychrophilic/mesophilic and thermophylic laccases available on public databases, no relationships were found between them and their temperature performance, unlike that described by Rouling, 2022. In this way, the correlation between amino acid content and chemical parameters observed for Antarctic laccases and their activity as a psychrophilic laccase should be revisited. Moreover, specific residue configurations around the active site of LcOe 229, cryptic at the moment, may be behind their psychrophilic activity.

Our results show that LcOe works as a psychrophilic enzyme and it has been proposed that psychrophilic enzymes show specific features that allow them to function at cold temperatures. One of them is that they show a less compact conformation than mesophilic and thermophilic proteins to allow frequent micro-unfolding events, which confer flexibility and plasticity for substrate accommodation at lower energy costs [40,41,42]. Indeed, the model structure of Antarctic laccases show a very long linker D2–D3 rich in Met that can provide structural flexibility and which is important for this activity and additional copper binding. The structure of LcOe 229 lacks the long linker D2–D3 and shows a compact structure similar to their mesophilic homologous Pa5930 and Pp4816. The use of laccases in bioremediation treatments in open environments, where there is no temperature control (such as those used for the decontamination processes of textile dyes, antibiotics, aromatic xenobiotics, etc.), as well as in food industry processes, where low to moderate heating is applied to preserve thermolabile components, requires microbial laccases that would perform their oxidative role better than mesophilic or thermophilic ones [6,7,20].

Finally, our structural studies have revealed high structural similarity between LcOe 229 and LAB laccases Pa5930 and Pp4816 previously characterized by our group, having an extended N-terminal and C-terminal end as well as two conserved Met residues at the entrance of the T1Cu site [11]. The presence of Met residues close to the T1Cu site is observed in the structures of CueO and Tth-MCO involved in metal ion binding, such as Ag^+^ and Cu^2+^, thus, we propose that these two Met in LAB laccases could be involved in the coordination of such metals. Moreover, we have trapped, in one of the crystals of LcOe 229, the imidazole group of a His residue located at the entrance of the T1Cu close to the two conserved Met residues as if occupying a substrate binding site. The aromatic nature of the imidazole group could emulate the aromatic nature of another aromatic ring, indicating that LAB laccases and LcOe 229 have a different substrate binding site than CotA.

## 4. Material and Methods

### 4.1. Materials: Chemicals and DNA and Protein Purification Systems

The following chemicals and reagents were acquired from specific suppliers: 2,2-azino-bis(3-ethylbenzothiazoline-6-sulfonic acid) (ABTS), 2,6-dimethoxyphenol (2,6-DMP potassium ferrocyanide (K_4_[Fe(CN_6_)]), tyramine, dopamine, phenylethylamine, putrescine, N-(3-dimethylaminopropyl)-N′-ethylcarbodiimide (EDC), 1,10-phenanthroline, 2,2′-bipyridyl, clorgyline, cyclopropylamine, deprenyl, pargyline HCl, rasagiline, semicarbazide, NaCl, NaF, isopropyl-β-D-thiogalactopyranoside (IPTG), arabinose, kanamycin, chloramphenicol, and molecular weight standard proteins were obtained from Sigma-Aldrich (Madrid, Spain). Nickel-chelating nitrilotriacetic acid (Ni^2+^-NTA) agarose was acquired from Qiagen (Hilden, Germany). All other chemicals and reagents used were of analytical grade. Restriction enzymes were obtained from New England Biolabs (Beverly, MA, USA), T4 DNA ligase from Roche Diagnostics (Barcelona, Spain), and DNA polymerase from Invitrogen (La Jolla, CA, USA). The UltraClean^®^ PCR Clean-Up kit (Metabion, Planegg, Germany) and the NZyMiniprep kit (Nzytech, Lisbon, Portugal) were utilized. DNA synthetic oligonucleotides were procured from Eurofins MWG Operon (Ebersberg, Germany).

### 4.2. Strains, Enzymes, and Plasmids

The *O. oeni* 229 strain was obtained from the Enolab collection and cultivated in MLO medium at a temperature of 28 °C following the method described by Caspritz and Radler [43]. For manipulation, amplification, and propagation of the LcOe 229 encoding gene, *Escherichia coli* DH5α [F^−^ φ80Δ*lacZ*M15 Δ(*lacZYA-argF*)U169 *deoR recA1 endA1 hsdR17 supE44* λ-*thi*-1 *gyrA*96 *relA*1] was used as a host. The overexpression vector employed was the pET-28a(+) plasmid (Novagen, Madison, WI, USA). *E. coli* BL21(DE3) [F^−^ *ompT hsdSB*(r_B_^−^m_B_^−^) *gal dem* (*λIts857 indI Sam7 nin5 lavUV*5*-*T7*gene*1)], which also carried the pGro7 plasmid, was utilized as the host for expressing the gene of interest under the control of the T7 promoter [11]. The pGro7 plasmid, designed to facilitate the efficient expression of multiple chaperones (groES-groEL) that collaborate in the protein-folding process [44], was employed for the coexpression of the LcOe 229 protein. *E. coli* cells were grown at 37 °C in Luria–Bertani (LB) medium, while transformants, when necessary, were cultured in LB medium supplemented with kanamycin (50 μg mL^−1^) or kanamycin plus chloramphenicol (20 μg mL^−1^). Genomic DNA was purified using the Ultra Clean™ Microbial DNA Isolation Kit, plasmid DNA was isolated using the Ultra Clean™ 6 min mini plasmid prep Kit, and PCR products were purified using the Ultra Clean™ PCR Clean-Up Kit, all of which were obtained from MoBio (Carlsbad, CA, USA). DNA synthetic oligonucleotides were purchased from EurofinsMWG Operon (Ebersberg, Germany), and restriction enzymes were obtained from New England Biolabs.

### 4.3. Cloning of the Gene Encoding the LcOe 229 Laccase, and Construction of the Expression Plasmid

The laccase gene of LcOe 229 was amplified through PCR using specific primers designed for this purpose. The forward primer, LcOe1 (5′-GAT***GCTAGC***ATGGAATTAATCAAAAATTATTT-3′), and the reverse primer, LcaOe2 (5′-CCG***GGATCC***TTACATTCGCATATTCGAATC-3′), were used. The recognition sites for NheI and BamHI, located at the beginning and end of the coding sequence, are indicated in bold italics. The PCR and subsequent molecular manipulations followed the conditions described in Callejón, Sendra, Ferrer, and Pardo [10].

The recombinant laccase gene was then cloned into the pET-28a(+) plasmid, resulting in the construction of the pET-28a(+)-LcOe 229 plasmid, which was used to transform *E. coli* DH5α by electroporation. The amplified plasmid was extracted, purified, and cloned into *E. coli* BL21(DE3) carrying the pGro7 plasmid.

### 4.4. Recombinant Laccase Expression and Purification

BL21(DE3) cells were cultivated in 2 litres of LB broth supplemented with kanamycin (50 μg mL^−1^), chloramphenicol (20 μg mL^−1^), and 1 mM CuCl_2_. The culture was stirred at 200 rpm until a 0.6–0.8 O.D. was reached, and then 1 mM IPTG was added. After this addition, the culture was incubated at 20 °C with stirring at 120 rpm for four hours and then the stirring was turned off and the incubation extended overnight at the same temperature [10]. Cells were recovered by centrifugation, frozen at −80 °C, thawed, and mechanically lysed as in Callejón, Sendra, Ferrer, and Pardo [10]. Cell debris was removed by centrifugation at 3500 rpm for 15 min at 4 °C (Multifuge 1 S-R, Heraeus, Hanau, Germany). The supernatant was collected and loaded onto a Ni^2+^-NTA-agarose column (0.8 × 2.5 cm), previously equilibrated with equilibration buffer (50 mM sodium phosphate, 300 mM NaCl, 5 mM imidazole, pH 8.0). Non-retained proteins were washed out with five column volumes of equilibration buffer. The retained protein was eluted with elution buffer (equilibration buffer containing 250 mM imidazole). Fractions of 0.2 mL were collected and examined for laccase activity using the standard reaction buffer (SRB): 50 mM sodium acetate, 0.1 mM CuSO_4_, pH 4.0, using 2 mM ABTS as substrate [10,11]. Protein concentration of the fractions was calculated using Pierce^TM^ kit BCA (Thermo Scientific, Waltham, MA, USA).

Purified Histag-LcOe 229 fractions were digested with thrombin using the Thrombin CleanCleave kit (Sigma-Aldrich, Madrid, Spain) containing Thrombin-Agarose resin (50% suspension in 50% glycerol, 20 mM Tris-HCl, pH 8.2) and 10x Cleavage Buffer (500 mM Tris-HCl, pH 8.0, 100 mM CaCl_2_). The resin was added in a ratio of 100 μL of resin per 1 mg of LcOe with 100 μL of 10× Cleavage Buffer, brought to 1 mL with water, and then incubated overnight at 4 °C. The digested protein was recovered in the supernatant after centrifugation for 5 min at 500× *g*. Then, the supernatant was loaded into a HisTrap HP column (Cytiva Lifescience, Marlborough, MA, USA) to perform affinity chromatography. The column was equilibrated with five column volumes of buffer A (50 mM Tris pH 8.0 and 150 mM NaCl) and eluted with buffer B (buffer A containing 200 mM imidazole). The digested protein eluted in the flowthrough as it did not bind to the HisTrap resin, and then, it was further purified with a size exclusion chromatography using a ProSEC 16/60 6-600 HR column (Generon, Houston, TX, USA) equilibrated in buffer A. Protein fractions were concentrated to 20 mg/mL, frozen with N_2_(l), and stored at −80 °C.

### 4.5. Biochemical Characterization of LcOe 229 Laccase

The UV–Vis absorption spectrum (300–800 nm) of the LcOe 229 pure recombinant laccase was performed as described in Callejón, Sendra, Ferrer, and Pardo [10]. The relative molecular mass of the denatured protein was determined in a polyacrylamide gel electrophoresis (PAGE) SDS-7.5% by comparison with Page Ruler Unstained Marker from Thermo Scientific (Madrid, Spain).

Kinetic parameters of LcOe 229 laccase were determined using different concentrations of ABTS (0.01–2 mM), 2,6-DMP (0.4–10 mM) and potassium ferrocyanide (0.075–2 mM) at room temperature (24 °C), as described in Olmeda, Casino, Collins, Sendra, Callejón, Huesa, Soares, Ferrer, and Pardo [11]. Reactions were carried out in a total volume of 200 µL using 50 mM McIlvaine buffer at pH 2.5, 4 and 8 for ABTS, potassium ferrocyanide, and 2,6-DMP, respectively, at least in triplicates. Enzyme was added at 1.85 µg/mL into the ABTS assay, 94.4 µg/mL into the 2,6-DMP assay, and 18.88 µg/mL into the ferrocyanide assay. All reactions were followed by increases in OD_420_ for the ABTS and ferrocyanide and OD_468_ for the 2,6-DMP in 96-well microplate. The data analysis was performed with the option “Enzyme kinetics: velocity as a function of substrate” of the GraphPad Prism 10 software (GraphPad Software, Boston, MA, USA) and the enzyme specific activity was expressed in units (U/mg), defined as μmol of substrate oxidised min^−1^ and mg^−1^ of protein. The extinction coefficients used, corresponding to the oxidation products, were as follows: ABTS, ε_420_ = 36,000 M^−1^ cm^−1^; K_4_[Fe(CN_6_)], ε_420_ = 880 M^−1^ cm^−1^; and 2,6-DMP, ε_468_ = 14,800 M^−1^ cm^−1^ [11].

To determine the effect of pH on laccase activity with ABTS, potassium ferrocyanide, and 2,6-DMP different buffers were used to cover a 1–9 pH range. To get pH values from 1–2, a KCl-HCl 50 mM buffer was used; to cover the range 2.5–9, a 50 mM C_6_H_8_O_7_–Na_2_HPO_4_ buffer (McIlvaine buffer) was employed. All of them contained 0.1 mM CuSO_4_ and one of the substrates (2 mM ABTS, 2 mM potassium ferrocyanide, or 4 mM DMP). The laccase concentration used was 2.78 µg/mL for every substrate. Enzyme activity on these substrates was determined spectrophotometrically at an OD_420_ for ABTS and potassium ferrocyanide, and at an OD_468_ for 2,6-DMP after 30 min incubation.

The effect of temperature on LcOe 229 laccase activity in the range 0 to 100 °C was checked by measuring ABTS oxidation using 200 µL McIlvaine buffer pH 2.5 with 0.1 mM CuSO_4_ and 2 mM ABTS to which 1 µL of an enzyme solution of (1.39 µg mL^−1^) was added. The reaction medium was pre-incubated for 5 min at the corresponding temperature before the addition of the recombinant enzyme, and then proceeded at that temperature. For thermal stability analysis, 200 μL of McIlvaine buffer pH 2.5 plus 0.1 mM CuSO_4_ and 1.39 µg mL^−1^ of enzyme was pre-incubated at different temperatures ranging from 28 to 100 °C for 5 min. After recovering the room temperature, reactions were initiated by the inclusion of ABTS at the final concentration of 2 mM. In temperature and thermal stability analyses, reactions were developed for 3 min then stopped by adding sodium azide to a final concentration of 1 mM. Oxidation of ABTS was measured by recording the increase in OD_420_ in microplate wells.

Different potential inhibitors were tested on the laccase LcOe 229 enzymes. These chemical compounds were: 2,2′-bipyridyl, EDTA, clorgyline, cyclopropylamine, deprenyl, EDC, pargyline, phenanthroline, rasagiline, semicarbazide, sodium azide; sodium fluoride, sodium chloride, cysteine HCl monohydrate, thioglycolic acid, and zinc chloride, all at 1 mM concentration. After incubating 10 μL of a 2.78 µm L^−1^ enzyme solution in McIlvaine buffer pH 2.5 with 0.1 mM CuSO_4_ and 1 mM inhibitor, reaction was initiated by the addition of ABTS (2 mM final concentration) and left to progress for 3 min, then was stopped by adding 1 mM sodium azide to stop the reaction. Oxidation of ABTS was measured by recording the increase in OD_420_ in microplate wells. The value from a reaction without inhibitor was considered as 100% relative enzyme activity (control).

### 4.6. Oligomeric State Determination by SEC

Size exclusion chromatography experiments were conducted by injecting 100 μg of Histag-LcOe 229 in 50 µL into a Superdex 200 3.2/300 (Cytiva Lifescience, Marlborough, MA, USA) resolutive column equilibrated with two column volumes of buffer A at a flow rate of 0.05 mL/min. The elution of the protein was followed with a UV detector at 280 nm. The apparent molecular weight was calculated from a calibration curve calculated using standards.

### 4.7. Protein Crystallography

Crystals were obtained using the sitting drop vapour diffusion technique. Crystallization of Histag-LcOe 229 was achieved by mixing 0.3 µL of a solution containing 20 mg/mL of protein and 50 µM of CuCl_2_ with 0.3 µL of different screening solutions (JBScreen Classic HTS I and HTS II, Jena Bioscience, Jena, Germany). Crystals were grown in a condition with 50% MPD, thus, they were harvested and frozen directly as this condition serves as a cryoprotectant. Crystals diffracted X-rays at 3.5 Å. Crystallization of LcOe 229 was achieved by mixing 0.3 µL of a solution containing 13 mg/mL of protein and 50 µM of CuCl_2_ with 0.2 µL of the same screening solutions. Crystals were grown in a condition containing 18% PEG 10000, 20% glycerol, 100 mM Tris-HCl pH 8.5, and 100 mM NaCl. Crystals were harvested and frozen directly as this condition also serves as cryoprotectant, then, they diffracted X-rays to 3 Å. Diffraction and data collection for the crystals was conducted in the BL13-XALOC of Alba Synchrotron (Cerdanyola del Vallès, Spain). Datasets of the highest resolution were used to solve the structures. Data integration was performed with XDS [45] and data reduction was performed with Aimless, present in the CCP4 suite [46]. Molecular replacement was conducted with Phaser [47] using as a template an Alphafold2 [48] predicted structure. The final structural models were obtained by iterative cycles of tracing with Coot [49] and refinement with Refmac5 [50]. The Ramachandran plot for refined Histag-LcOe 229 showed 92.62% residues in favoured regions, 7.07% in allowed regions, and 0.32% of outliers. For LcOe 229, the Ramachandran plot showed 93.7% residues in favoured regions, 6.21% in allowed regions, and 0.21% of outliers.

## Figures and Tables

**Table 1:** Kinetics parameters of LcOe 229 recombinant laccase for the substrates 2,2-azino-bis(3-ethylbenzothiazoline-6-sulfonic acid) (ABTS), 2,6-dimethoxyphenol (2,6-DMP) and potassium ferrocyanide K_4_[Fe(CN_6_)].

Substrate	Km (mM)	Vmax (U/mg)	Kcat (s^−1^)	Kcat/Km (mM^−1^ s^−1^)
ABTS	0.07 (±0.00) ^a^	12.06 (±0.39)	11.20 (±0.38)	161.67
2,6-DMP	0.73 (±0.17)	0.01 (±0.00)	0.01 (±0.00)	0.01
Ferrocyanide	1.14 (±0.14)	11.10 (±1.16)	10.27 (±1.07)	9.02

^a^ LcOe 229 showed sigmoidal kinetic with ABTS. For this substrate, the K_0.5_ substrate concentration that generates a half-maximal velocity and is operationally similar to the Michaelis constant, Km was determined.

**Table 2:** Data collection and refinement statistics for the obtained structures.

	Histag-LcOe 229	LcOe 229
**Data collection**		
Space group	C 1 2 1	P 1 2 1
Cell dimensions		
a, b, c (Å)	206.26, 56.69, 106.05	52.75, 97.96, 96.34
α, β, γ (°)	90.00, 118.03, 90.00	90.00, 91.61, 90.00
Resolution (Å)	93.61–3.50 (3.83–3.50)	68.55–3.00 (3.18–3.00)
No. reflections	93,984 (22,297)	90,396 (14,384)
Rsym or Rmerge	0.419 (1.135)	0.273 (0.715)
Rpim	0.262 (0.715)	0.199 (0.532)
I/σI	4.8 (2.0)	4.4 (2.3)
Completeness (%)	99.9 (100)	97.8 (97.8)
**Refinement**		
Rwork/Rfree	0.21/0.26	0.21/0.26
No. atoms		
Protein	7348	7405
Ligand/Ion	8	8
Water	11	74
B-factors		
Protein	67.1	20.88
Ligand/Ion	94.29	24.6
Water	38.54	6.88
R.m.s deviations		
Bond lengths (Å)	0.004	0.005
Bond Angles (°)	1.067	1.132
PDB Code	9F3Z	9F1T

**Table 3:** Chemical and enzymatic parameters for several laccases from psychrophilic, mesophilic, and thermophilic organisms.

Organism Type	Laccase	pI	Mw (Da)	Enzymatic Optimal T (°C)	Activity at 20 °C (%)	Total Aas	M	G	P	R	C	D	E	N	Q	Aliphatic Index
							%	%	%	%	%	%	%	%	%	
Antarctic Gram−	Hmer-R1t3	5.70	67373.47	??		607	5.9	10.0	5.6	7.2	0.2	5.8	6.9	3.1	3.5	75.1
	Hpie-resA	5.65	67402.35	??		607	6.1	10.2	5.4	7.2	0.2	5.4	7.2	3.3	3.6	72.9
	Hcol-FME66	5.51	67397.35	??		607	6.3	10.2	5.6	7.2	0.2	5.6	7.1	3.5	3.6	72.5
	PhaMOx	5.47	64212.83	35		576	6.4	7.5	5.4	5.2	0.2	8.0	4.5	4.3	3.5	75.3
Mesophylic Gram−	Ecol-6898	6.07	53420.43	55		488	5.7	10.2	6.8	7.8	0.6	6.1	4.3	3.5	3.6	83.1
Mesophilic Gram+	Bsub-6077	5.91	58498.99	75		513	1.6	6.6	9.4	5.7	0.8	6.6	7.2	3.8	2.5	77.9
LAB Gram+	Ppar-3909	5.11	58216.56	28	93	511	4.1	7.6	6.8	3.1	1.0	9.2	5.9	4.3	4.1	71.1
	Llac-5298	5.32	58613.52	28	86	520	4.2	7.9	7.9	3.7	0.8	8.1	5.8	3.1	3.5	74.2
	Ppen-4816	5.37	57312.97	55	25	509	3.9	7.9	7.9	3.7	0.8	8.3	5.5	2.9	3.7	73.7
	Lpar-4314	5.37	57298.94	28	57	509	3.9	7.9	7.9	3.7	0.8	8.4	5.3	2.9	3.7	73.7
	Lpla-J16	4.93	56875.9	60	20	501	3.4	7.0	8.0	4.4	1.0	10.2	5.4	3.8	4.4	71.5
	Paci-5930	5.42	54361.44	28	95	477	3.1	7.8	7.1	4.0	0.8	7.8	6.1	5.7	4.2	79.5
	Lsak-LS	5.37	57327.00	25	84	509	3.9	7.9	7.9	3.7	0.8	8.3	5.5	2.5	3.1	73.9
	Ooen-2291	5.70	55544.12	10	70	488	4.1	7.4	6.6	4.3	0.8	8.4	5.3	4.8	2.6	80.5
Thermophylic Gram−	Tth-MCO	7.09	48855.73	95		440	3.0	8.4	10.0	7.3	0.2	4.1	6.8	1.6	2.3	96.6

pI: theoretical isoelectric point; MW: molecular weight; enzymatic optimal temperature; retained activity at 20 °C; amino acid content in % for M, G, P, R, C, D, E, N, and Q; aliphatic index; ?? stands for unknown. The colours in the boxes represent the lowest (blue) and highest (red) values for each respective column.

## Data Availability

The X-ray crystallographic coordinates reported for all the structures have been deposited at the Protein Data Bank. For LcOe 229 and Histag-LcOe 229, the PDB accession codes are 9F1T and 9F3Z, respectively.

## Supplementary Materials

The following supporting information can be downloaded at: https://www.mdpi.com/article/10.3390/ijms25158521/s1.
