# Supplementary material for: Biochemical and Structural Characterization of a Novel Psychrophilic Laccase (Multicopper Oxidase) Discovered from Oenococcus oeni 229 (ENOLAB 4002)
Source: Int J Mol Sci. 2024 Aug 5;25(15):8521. doi: 10.3390/ijms25158521 (PMC11312515; doi:10.3390/ijms25158521)
Supplement: Supplementary file 1 [file ijms-25-08521-s001.zip › ijms-3083496-supplementary.pdf]

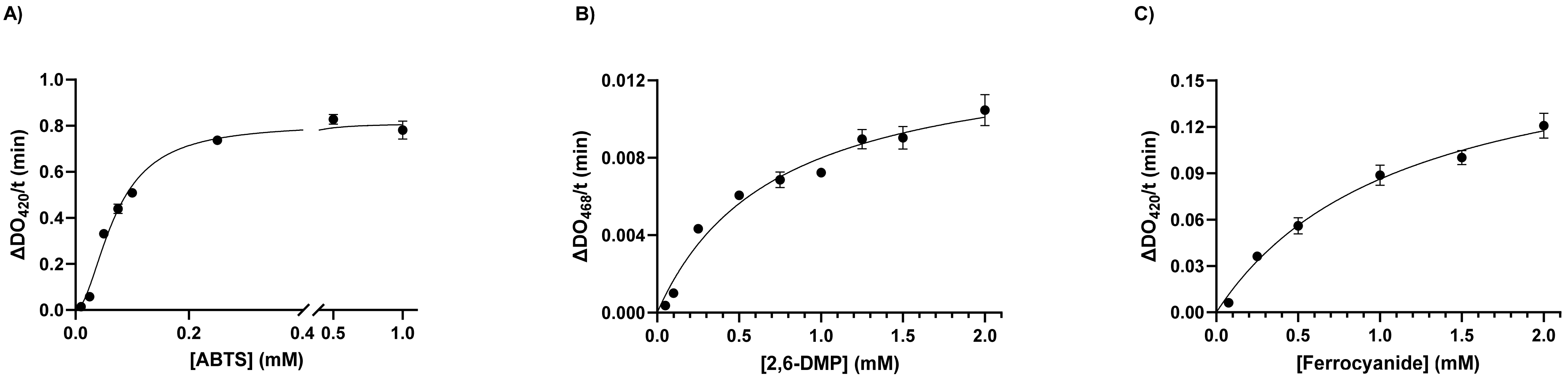

Supplementary Figure S1. Kinetic analysis of recombinant laccase LcOe 229 with the substrates 2,2-azino-bis(3-ethylbenzothiazoline-6-sulfonic acid) (ABTS) (A), 2,6-dimethoxyphenol (2,6-DMP) (B) and potassium ferrocyanide $K_4[Fe(CN_6)]$ (C). The reaction rates were obtained from the linear portion of the progress graph and were plotted against the corresponding substrate concentrations. The kinetic data were fitted by nonlinear regression to an empirical sigmoid equation for ABTS and to a Michaelis-Menten equation for 2,6-DPM and ferrocyanide with the GraphPad Prism 10 software. The tests were performed in triplicate.

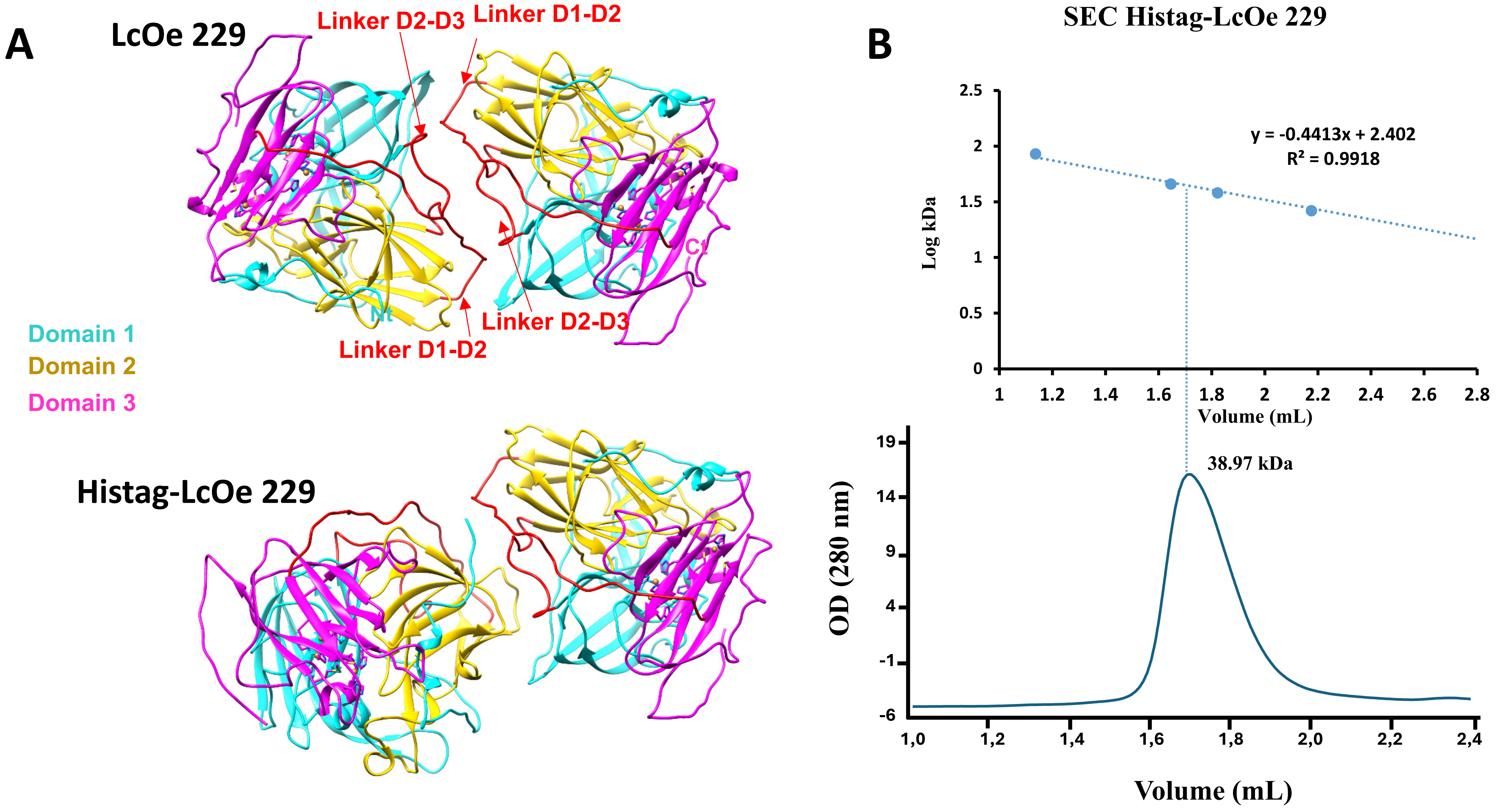

Supplementary Figure S2. Evaluation of the quaternary structure of LcOe 229. A) Structures of LcOe 229 and Histag-LcOe 229 shows two molecules in the asymmetric unit that are formed by crystal packing. B) Size-exclusion chromatography (SEC) of Histag-LcOe 229 elutes in a volume that corresponds to a molecular weight of monomer interpolated in the calibration curve obtained for the column.

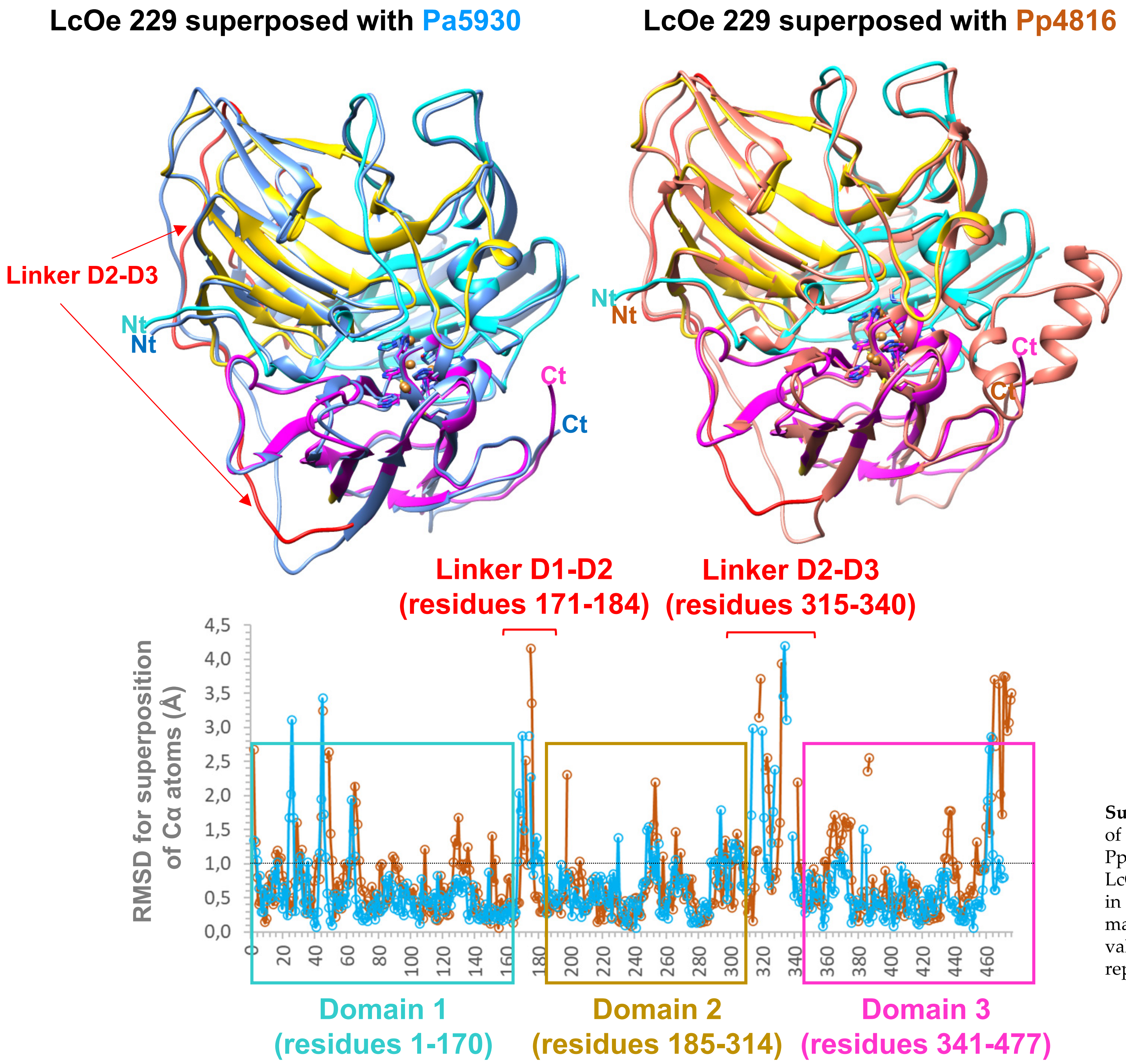

Supplementary Figure S3. Structural superposition of LcOe 229 with Pa5930 (in blue; PDB: 6Z0K) and Pp4816 (in orange; PDB: 6XJ0). The structure of LcOe 229 has been colored by domains (Domain 1 in cyan, Domain 2 in yellow and Domain 3 in magenta). The root mean square deviation (RMSD) values for the Cα atoms in the superposition is represented versus the number of residues.

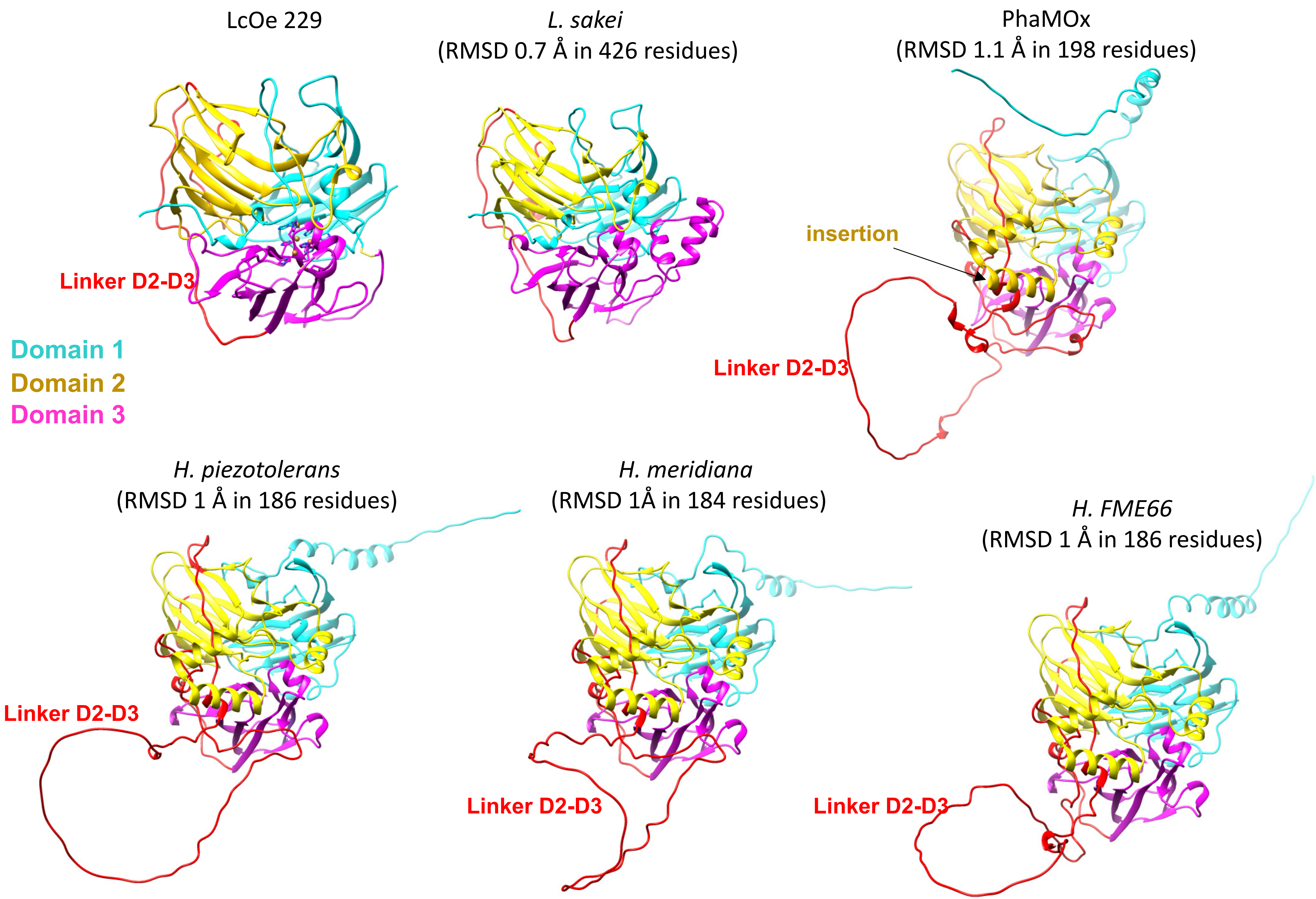

Supplementary Figure S4. Structure of LcOe 229 compared to the model structures of *L. sakei* and Antarctic species. The model structures have been obtained with AlphaFold2. The domains have been color-coded (Domain 1 in cyan, Domain 2 in yellow and Domain 3 in magenta) highlighting the linker D2-D3 in red.

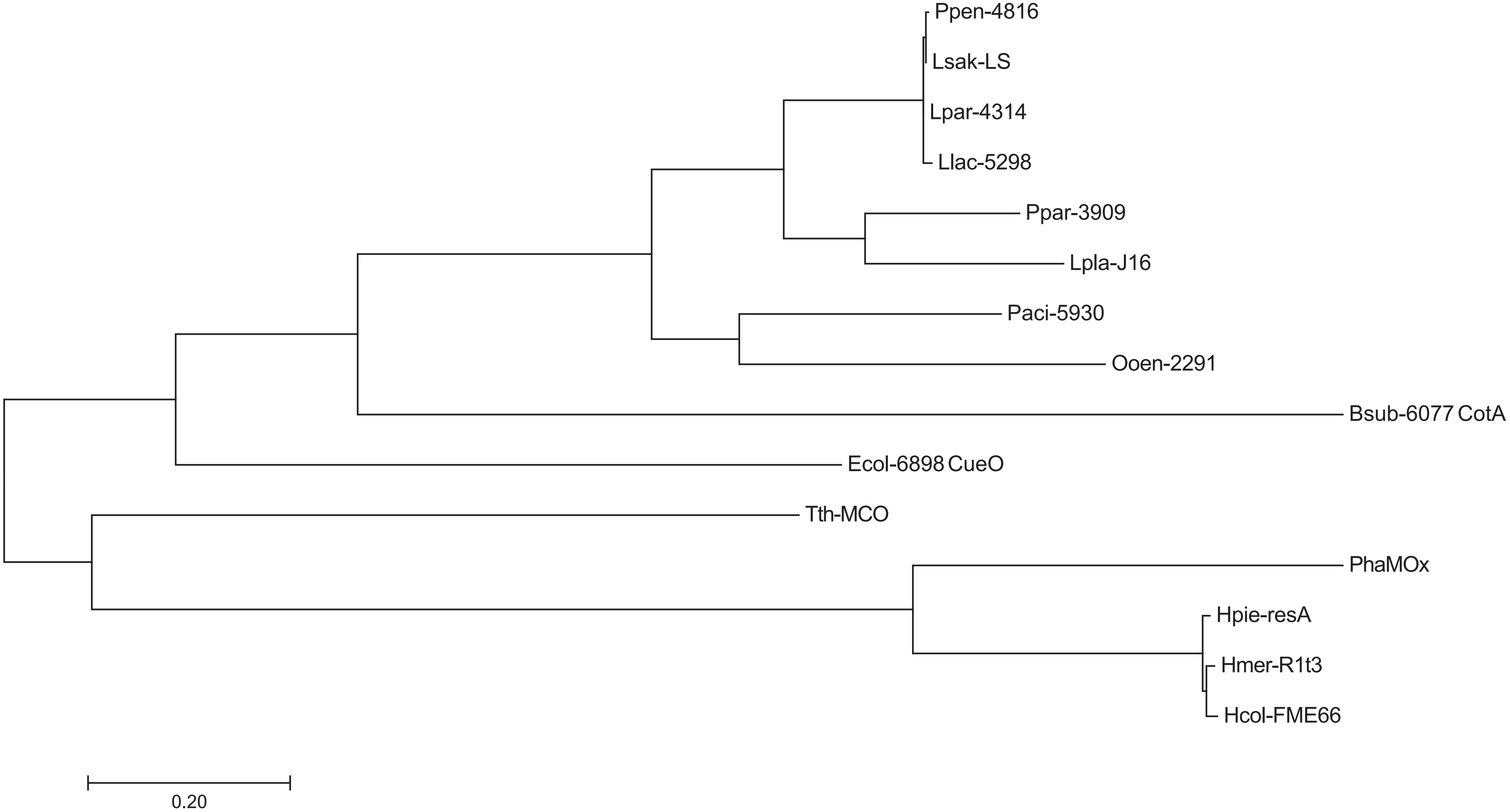

Supplementary Figure S5. Molecular Phylogenetic analysis by Maximum Likelihood method

The evolutionary history was inferred by using the Maximum Likelihood method based on the JTT matrix-based model [38]. The tree with the highest log likelihood (-7475.89) is shown. Initial tree(s) for the heuristic search were obtained automatically by applying Neighbor-Join and BioNJ algorithms to a matrix of pairwise distances estimated using a JTT model, and then selecting the topology with superior log likelihood value. The tree is drawn to scale, with branch lengths measured in the number of substitutions per site. The analysis involved 15 amino acid sequences. All positions containing gaps and missing data were eliminated. There were a total of 377 positions in the final dataset. Evolutionary analyses were conducted in MEGA7 [39].
